# Encapsulation of Capacitive Micromachined Ultrasonic Transducers (CMUTs) for the Acoustic Communication between Medical Implants

**DOI:** 10.3390/s21020421

**Published:** 2021-01-09

**Authors:** Jorge Oevermann, Peter Weber, Steffen H. Tretbar

**Affiliations:** Ultrasound Department, Fraunhofer-Institute for Biomedical Engineering, 66280 Sulzbach, Germany; peter-karl.weber@ibmt.fraunhofer.de (P.W.); steffen.tretbar@ibmt.fraunhofer.de (S.H.T.)

**Keywords:** acoustic communication, biomedical communication, CMUT, medical implants

## Abstract

The aim of this work was to extend conventional medical implants by the possibility of communication between them. For reasons of data security and transmitting distances, this communication should be realized using ultrasound, which is generated and detected by capacitive micromachined ultrasonic transducers (CMUTs). These offer the advantage of an inherent high bandwidth and a high integration capability. To protect the surrounding tissue, it has to be encapsulated. In contrast to previous results of other research groups dealing with the encapsulation of CMUTs, the goal here is to integrate the CMUT into the housing of a medical implant. In this work, CMUTs were designed and fabricated for a center frequency of 2 MHz in water and experimentally tested on their characteristics for operation behind layers of Polyether ether ketone (PEEK) and titanium, two typical materials for the housings of medical implants. It could be shown that with silicone as a coupling layer it is possible to operate a CMUT behind the housing of an implant. Although it changes the characteristics of the CMUT, the setup is found to be well suited for communication between two transducers over a distance of at least 8 cm.

## 1. Introduction

Medical implants have been used successfully for a long time for the treatment of various diseases. In addition to autonomous implants, like pacemakers or cochlear implants, therapeutic approaches are also conceivable which require the interaction of several implants provoking the desired reaction at different points within the body. For example, Wegmueller et al. mentioned the possibility of a pacemaker that adapts its function to vital parameters recorded in different regions of the body [1]. Another example is scoliosis therapy. A large number of interacting implants along the spinal column could have a stimulating or detonating effect on the respective muscles at the crucial points in accordance with the current posture (e.g., standing or sitting) to minimize and permanently eliminate malpositions. In order to enable a coordinated reaction of implants, a stable communication system between them is crucial. According to V. K. Khanna, communication and power supply for medical implants can be provided either by percutaneous leads or by wireless technologies [2]. The former show various disadvantages, including an increased risk of infection, low acceptance by the patient, and limitations of mobility. Therefore, the preferred wireless methods are mainly based on either acoustic (ultrasound) or electromagnetic waves. One disadvantage of using electromagnetic waves is the low penetration depth, which Bos et al. state at only 5 cm [3]. In addition, these methods would have lower transmission rates of only 1.56 Mbps [3] compared to ultrasound with up to 28.12 Mbps [4] for data transmission. In addition to the first applications in the biomedical field, ultrasound is established as a communication tool especially for underwater applications [5]. According to Melodia et al., the two alternatives here, radio-frequency as well as optical waves are not suitable for long distances, hence ultrasound finds favor [5]. Radio-frequency electromagnetic waves would only propagate over long distances at very low frequencies, requiring large antennas. Optical waves instead would fail due to scattering and the need for highly accurate alignment of the laser beam. In order to minimize the weaknesses of underwater communication based on ultrasound, such as limited and depth-dependent bandwidth or multi-path propagation, efforts are made to improve transmission technology and communication protocols. Since human tissue also mainly consists of water, it is reasonable to assume that ultrasound can also be used for data transmission in the biomedical field. Within this project, the communication is therefore decided to be realized on an acoustic basis with the help of ultrasonic waves. In terms of data security, it is advantageous that the ultrasonic waves hardly leave the human body due to the large acoustic impedance difference between tissue and air. This makes it much more difficult to intercept the data.

In the last decades, considerable progress has been made in the field of capacitive micromechanical ultrasonic transducers, especially with regard to fabrication quality and predictability of characteristic parameters [6]. In contrast to conventional ultrasonic transducers, for which the piezoelectric ceramic PZT is usually used, capacitive micromachined ultrasonic transducers (CMUTs) have the advantage that they can be manufactured RoHS compliantly. Apart from this, they offer an inherently wide bandwidth [7], a low self-heating [8], and great freedom in the selection of the transducer geometries that can be manufactured, because each transducer element is composed of a large number of single CMUT-cells. At the same time, this enables the transducers to be miniaturized [9]. For these reasons, CMUTs were chosen for the generation and detection of ultrasonic waves.

While there are many research groups that are intensively engaged in the study of CMUTs in general, relatively few are concerned with the application of CMUTs in the human body. Medical applications in the body require an encapsulation of the CMUT to protect the patient from the applied voltages, and to protect the CMUT from the surrounding tissue and mechanical damage. There are essentially only two approaches for this in the literature: Either the CMUT is covered with a layer of Polydimethylsiloxane (PDMS) or one of Parylene-C. Both materials are biocompatible. Parylene-C was used by Zhuang et al. [10] and Hsu et al. [11] in the form of a 2 µm thick protective layer on the CMUT-membrane. Zhuang et al. [10] state the period for which the Parylene-C coating ensured electrical insulation in an aqueous solution as 14 days. Experiments with PDMS as encapsulation material were performed by Moini et al. [12] (t = 100 µm), Jang et al. [13] (t = 150 µm), Chang et al. [14] (PDMS as lens material with a minimum thickness of 1.42 mm), Nikoozadeh et al. [15] (t = 180 µm), and Zhuang et al. [8] (t = 5 µm). Due to the small thickness and the Youngs modulus of the materials used for the protective layer, both materials changed the properties of the CMUT only slightly. Lin et al. [16] demonstrated that an encapsulation using a 150 µm thick layer of PDMS preserves the collapse voltage and alters the center frequency by only 5%. In a publication by la Cour et al., who have encapsulated their CMUTs with an approx. 900 µm thick layer of PDMS, a frequency shift through the encapsulation from 4.5 MHz to 4.1 MHz (9%) in transmitting and from 4.4 MHz to 3.9 MHz (11%) in receiving is determined [17]. In the same study, a 27% reduction in transmission pressure and a 35% decrease in receiving sensitivity is reported. While these materials promise sufficient insulation for short-term use, their long-term stability is uncertain.

A different approach was published by Zhang et al., using silicone oil together with a foil of polyurethane [18]. Due to its low water permeability, polyurethane is used to isolate the CMUT from the surrounding water, while silicone oil is used as a filler to prevent air from being trapped between the CMUT and polyurethane. In the paper described here, however, the effects of the encapsulation are neither investigated nor optimized, so that it can only be seen as a starting point for further work.

For medical implants, stable hermetic encapsulation is crucial. Titanium is often used for the encapsulation (or housing) [19], as it has been demonstrated that it can protect the human body from the inner parts of the implant over a long period of time and vice versa. Another promising material for the housing of implants is Polyether ether ketone (PEEK) [20]. For this reason, these two materials were investigated for the housing of the CMUT in this work. A silicone (Wacker Elastosil E43, [21]) was used as a coupling layer between the membrane and the housing of the implant. While the previous approaches to protect CMUTs used polymer layers, this is the first attempt to operate a CMUT in combination with PEEK and titanium, two materials established in the encapsulation of biomedical implants. For a better classification of the encapsulation method proposed in this paper, an additional CMUT array chip was coated with 5.5 µm thick Parylene-C so that a direct comparison with the state-of-the-art for general CMUT encapsulation is possible.

## 2. CMUT Simulation and Experimental Setup

**Figure 1:**
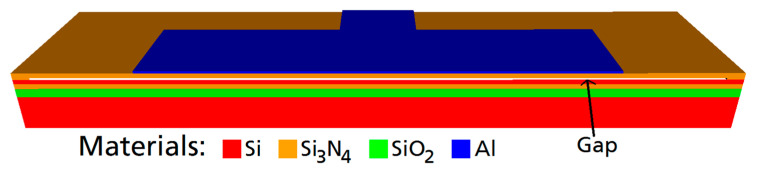
Capacitive micromachined ultrasonic transducer (CMUT) cell model simulated in OnScale.

First, the CMUT was designed. The objective was a center frequency of 2 MHz in water. An additional constraint was a broad bandwidth to enable effective communication as well as a high output pressure. Using the finite element software OnScale (formerly PZFlex) [22], a standard cell design of a rectangular Si_3_N_4_ membrane with an embossed aluminum electrode was modified (see Figure 1.). A single CMUT cell was simulated exploiting two symmetry axes. Meshing was applied with 20 finite elements per 14 µm (a half-width of the aluminum electrode) with a minimum number of 4 elements per dimension within each material layer. The material parameters used in the simulation can be found in Table 1. The properties of the silicone were determined experimentally, those for PEEK were found in the work of Fitch et al. [23]. The other materials are included in the OnScale database [22].

The final CMUT cell design has the following dimensions: Edge length of the membrane: 80 µm × 40 µm. Membrane thickness: 850 nm (600 nm-Si_3_N_4_, 250 nm-Al). Gap-height: 200 nm.

**Figure 2:**
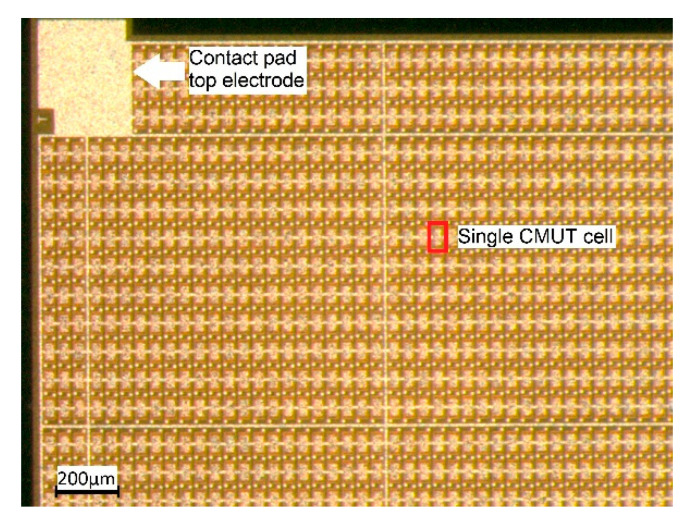
Section of the fabricated CMUT chip, edge length of a single membrane: 80 µm × 40 µm. membrane thickness: 850 nm (600 nm-Si_3_N_4_, 250 nm-Al). Gap-height: 200 nm.

The CMUT array chips were fabricated by microfab Service GmbH in Bremen, Germany, using a sacrificial release process (see [6] for a description of the process). Each CMUT array chip has edge lengths of 5 mm × 5 mm and is composed of 3416 CMUT cells connected in parallel. A photo of a section of such a CMUT chip is shown in Figure 2.

**Figure 3:**
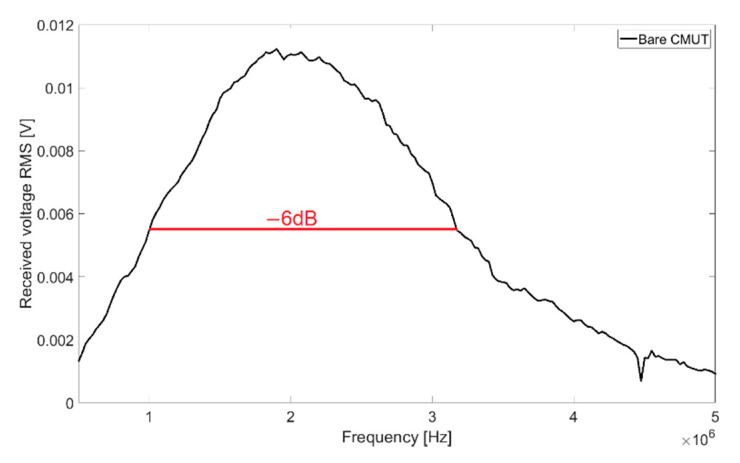
Frequency spectrum of an uncoated CMUT chip in Fluorinert FC-72, 60 V_DC_, 15 V_AC_, burst count 20.

With transmission measurements in Fluorinert FC-72, a full width at half maximum bandwidth (FWHM) of 102% and a central frequency of 2.08 MHz were determined. The resulting frequency spectrum for 60 V_DC_ bias voltage and 10 V_AC_ signal voltage is shown in Figure 3.

### 2.1. Medical Encapsulation

**Figure 4:**
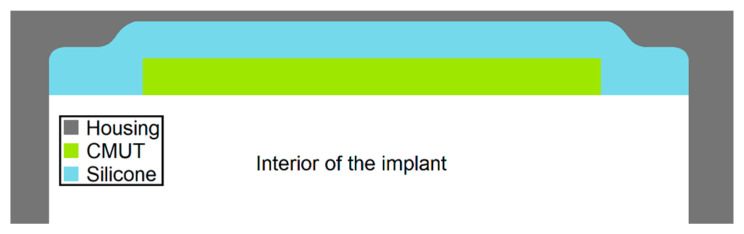
Schematic sketch of the CMUT chip inside the housing of the medical implant.

The idea of the encapsulation approach presented in this publication is to extend a conventional housing of a medical implant with a PEEK or titanium thickness of 500 µm by an acoustic window, which is either made of thinner titanium or alternatively of a PEEK foil (see Figure 4).

A protective layer with the thickness of a conventional implant encapsulation applied directly to the membrane would create a highly dampened composite oscillator. The approach presented here, to combine CMUTs with the protective layer of titanium (thickness 32 µm) or PEEK (thickness 25 µm), which simulates the housing of a medical implant, involves a layer of silicone between CMUT and the protective layers. The silicone serves as an adhesive and a coupling layer. The impedance discontinuities from CMUT to silicone, from silicone to PEEK/Titanium, and from PEEK/Titanium to tissue cause part of the sound energy to be reflected. Consequently, especially in the case of layer thicknesses in the order of the wavelength, interferences between the actual wave and reflected components occur within the respective layer. With longer signals (extreme case: CW excitation) standing waves can be induced. This can cause the output pressure wave to be amplified or attenuated.

The speed of sound in PEEK is about 2536 m/s [23]. In the frequency range of interest, from 500 kHz to 5 MHz, this corresponds to wavelengths between 5.07 mm and 507 µm. The speed of sound of titanium is 6100 m/s [22], resulting in wavelengths of 1.2 cm to 1.2 mm [24]. Since both of the foils examined are significantly thinner with layer thicknesses of 25 µm (PEEK) and 32 µm (titanium), interference within the layer is not to be assumed here. For the silicone used (Wacker Elastosil E43), a sound velocity of 1000 m/s was measured. The wavelengths in the frequency range of interest are located between 2 mm and 200 µm and the thickness of the applied silicone layer was 170 µm. In the frequency spectrum of the encapsulated CMUT, pressure maxima can be expected whenever the relative thickness of the silicone layer corresponds to an odd multiple of one-quarter of the wavelength of the signal in silicone. If the silicon layer thickness is an even multiple of half the wavelength, a standing wave is formed, and the emitted pressure is minimal.

### 2.2. Impedance Analyzer

An impedance analyzer (Keysight E4990A) was used to acquire the impedance spectra of the different CMUT setups. For comparability with the vibrometry measurements (Section 2.3), the impedance measurements for the basic characterization of the CMUT chips were carried out in air. The measurements were performed while applying bias voltages of 40 V_DC_, the maximum voltage provided by the impedance analyzer. The frequency was varied from 500 kHz up to 13 MHz, which is the limit of the test fixture used (Keysight 16047A). The AC voltage was 500 mV.

### 2.3. Laser Doppler Vibrometry

To examine the oscillation behavior of single CMUT membranes, experiments using a laser vibrometry system (Polytech UHF-120) were performed. CMUT chips were biased with 60 V_DC_ by Rohde and Schwarz DC Power Supply NGL 35. A function generator (Rohde and Schwarz AFGU) was connected to provide an AC signal of 15 V_PP_, using a bias tee (Picosecond 5530B). A single sine burst was used for excitation to find the resonance frequencies. To examine the steady-state behavior, continuous-wave signals were used. The investigated frequency range was from 500 kHz to 20 MHz. Laser vibrometry measurements were performed in air, since in the case of vibrometry measurements in Fluorinert, the refractive index and the height of the liquid must be precisely known and taken into account. An experimental setup for measurements in Fluorinert is currently being worked on. However, the difficulty remains that the height of Fluorinert has an influence on the result, but it is not known which height provides comparable conditions to the later applications in the human body or to the transmission experiments.

### 2.4. Transmit Experiments in Immersion

**Figure 5:**
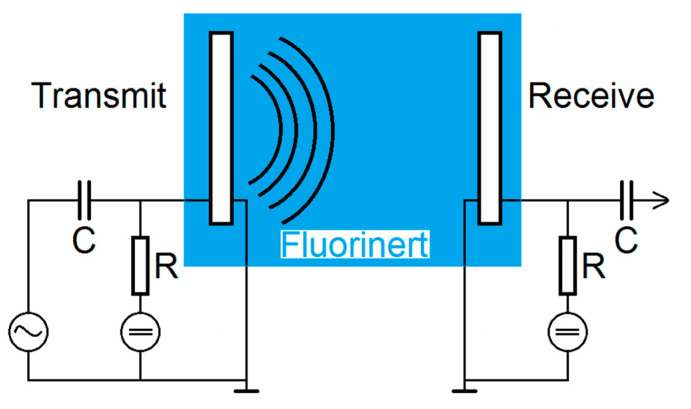
Schematic sketch of the setup for the transmit experiments.

The transmit-receive-properties of the CMUT array chips with different coatings were finally examined using a transmit setup in Fluorinert FC-72 (see Figure 5). Fluorinert FC-72 is an electrically insulating liquid with a density of 1.68 g/cm^3^ and a sound velocity of 512 m/s that was used to avoid short-circuits due to the exposed electrodes [25].

Using a Rohde and Schwarz DC Power Supply NGL 35, two CMUTs facing each other were biased individually by 60 V. One CMUT was used for transmitting ultrasonic signals, the second one to receive them. For excitation, a Rohde and Schwarz function generator AFGU was used to generate a sine burst N = 20 cycles with a signal amplitude of 10 V_PP_. The distance between transmitting and receiving CMUT was 83 mm. The received voltage was displayed and stored using a Keysight DSOX4024A Oscilloscope with a sample rate of 5 GSa/s. For a better signal-to-noise ratio, a 16-times averaging was used.

### 2.5. Examined CMUT Setups

**Figure 6:**
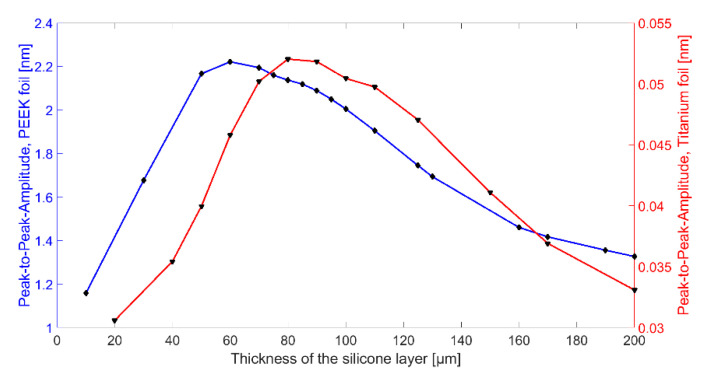
Simulated dynamic displacement of a polyether ether ketone (PEEK) foil (thickness 25 µm, blue curve) or a titanium foil (thickness 32 µm, blue curve) on top of a single CMUT cell with a silicone layer of different thickness, excited with 60 V_DC_ and a single sinusoidal burst at 2 MHz of 15 V_AC_.

After the optimization of the uncoated CMUT design, the combination of CMUT cell and coating was optimized by finite element analysis (FEA) to achieve a possibly high output power. For this purpose, the combination of CMUT cell and encapsulation was simulated with a single sinusoidal cycle excitation (2 MHz, 15 V_AC_, 60 V_DC_) for different silicone thicknesses. The resulting mean displacement of the PEEK foil is plotted in Figure 6. Depending on the silicone thickness, the resonance frequency of the entire system changes. For a frequency of 2 MHz, the maximum displacement could be determined for a silicone layer thickness of 60 µm for encapsulation with silicone and PEEK and for a silicone layer thickness of 80 µm for encapsulation with silicone and titanium.

**Figure 7:**
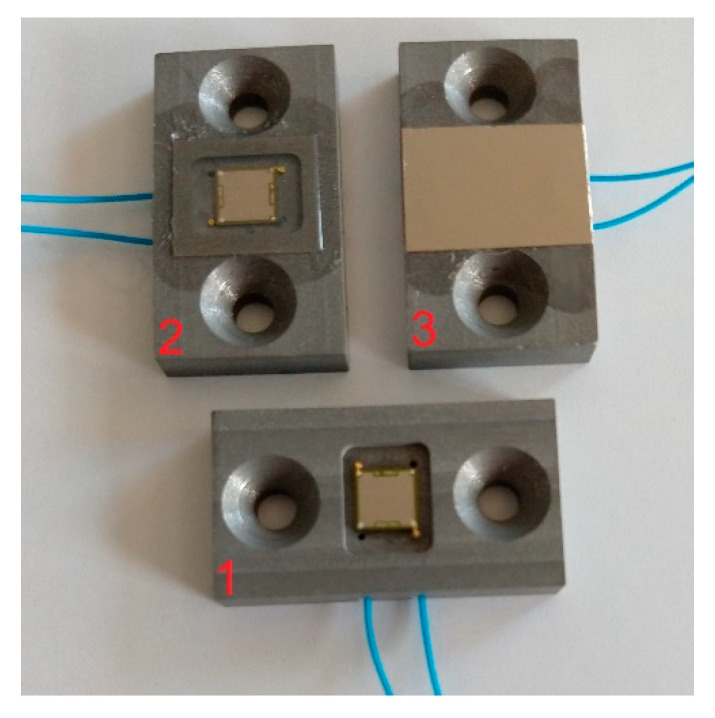
Setups validated in transmission experiments: (1) uncoated CMUT, (2) CMUT with silicone (t = 170 µm) and PEEK (t = 25 µm), (3) CMUT with silicone (t = 170 µm) and titanium (t = 32 µm).

Six different setups were examined experimentally (see examples in Figure 7):

- Uncoated CMUT without any protective layer (Chip 1)
- CMUT with 170 µm ± 5 µm thick silicone and 25 µm thickness PEEK foil on top (Chip 2)
- CMUT with 170 µm ± 5 µm thick silicone and 32 µm thick titanium foil on top (Chip 3)
- CMUT with 5.5 µm thick Parylene-C coating (Chip 4)
- CMUT with 50 µm ± 5 µm thick silicone and 25 µm thick PEEK foil on top (Chip 5)
- CMUT with 50 µm ± 5 µm thick silicone and 32 µm thick titanium foil on top (Chip 6)

According to the simulation results, CMUT chips with a silicone thickness of 60 µm for the encapsulation containing a PEEK foil and a silicone thickness of 80 µm for the encapsulation containing a titanium foil should be investigated experimentally. However, due to uncertainties in the coating process and shrinkage of the silicone during drying, a thickness of only 50 µm was determined for the applied silicone layers (Chip 5 and Chip 6). In addition, two CMUT chips (Chip 2 and Chip 3) were coated with a significantly thicker silicone layer (thickness 170 µm) in order to further investigate the influence of the silicone thickness.

The CMUT array chips had edge lengths of 5 mm × 5 mm. First, the CMUT array was bonded into a polyurethane-frame. The frame consisted of a rectangular recess in the middle, into which the array could be glued and which contained two cable feed-throughs for the connection cables. To the left and right of the recess, drilled holes were positioned for fixing the frame to the desired experimental setup. The recess depth was bigger than the thickness of the array chip so that it could easily be coated with silicone and a flush-mounted foil on top. The PEEK foil had to be plasma-activated for better adhesion on the silicone layer. This step was not necessary for titanium. After applying the foil, the silicon was left to dry for three days. During this process, the silicone shrinks a little so that the foil on top is sagging in the middle. The effects of this sagging have not been systematically studied. However, the directivity of the CMUT chips in the transmission measurements was not affected by this.

## 3. Experimental Characterization of the CMUT Chips

### 3.1. Impedance Measurements

**Figure 8:**
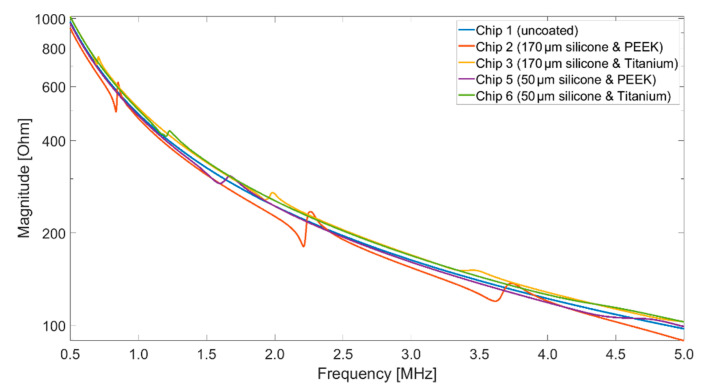
Electrical impedance in air for uncoated CMUT chips and for those covered with silicone and foil, 40 V_DC_.

In the first step, the impedance spectrum of the uncoated CMUT transducer was measured in air at a bias voltage of 40 V_DC_. In the frequency range between 500 kHz and 13 MHz (limited due to the setup used), no resonance was found for the uncoated CMUT array, as it was already in collapse mode. Due to the additional layers applied on the CMUT membrane, the collapse voltage was higher for the encapsulated CMUT chips, which is why the result shown in Figure 8 for the uncoated chip was still at a bias voltage of 40 V_DC_ for better comparability. After covering the CMUT array with silicone and PEEK or silicone and titanium, the measurement was repeated. The results are shown in Figure 8. Although the global behavior caused by the static capacitance of the array (C_S_ = 10.2 nF) did not change significantly, several resonances could be seen in the curves of Chip 2 and Chip 3. Chip 2 shows minima at 837 kHz, 2.21 MHz, 3.62 MHz, and 5.06 MHz. Chip 3 shows minima at 696 kHz, 1.93 MHz, and 3.38 MHz. Chip 5 shows a minimum at 1.59 MHz, Chip 6 shows a minimum at 1.20 MHz. The impedance measurements thus show that the CMUTs were not destroyed during the coating process and that additional resonance was induced by the coating.

### 3.2. Laser Vibrometry

The design of the CMUT cells was optimized with PZFlex for a resonance frequency of 2 MHz in Fluorinert FC-72, but the vibrometry setup used is only suitable for measurements in air.

**Figure 9:**
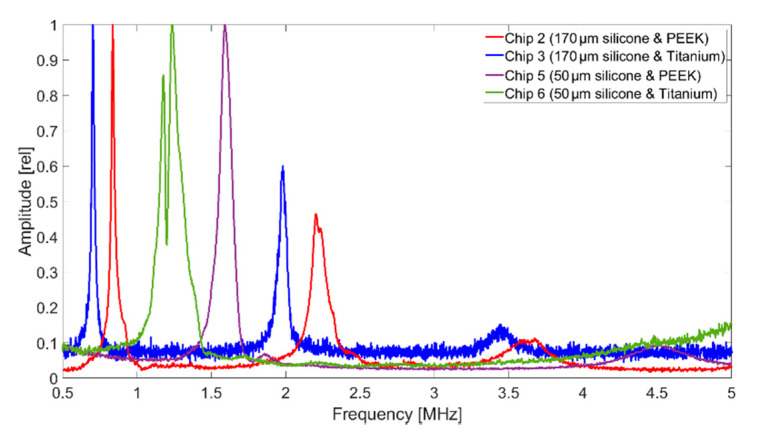
Frequency spectra by vibrometry of silicone and foil-coated CMUT chips in air, 60 V_DC_, 15 V_AC_, single sinusoidal burst at 3 MHz.

In the case of the CMUT chip 2 (coated with 170 µm silicone and PEEK), the vibrometry measurement in air showed resonances at 838 kHz, 2.20 MHz, and 3.69 MHz. Chip 3 (silicone and titanium) had resonance at 703 kHz, 1.98 MHz, and 3.5 MHz. Reducing the thickness of the silicone layer to approx. 50 µm, lead to a single resonance peak at 1.59 MHz for Chip 5 and two peaks at 1.18 MHz and 1.23 MHz for Chip 6. The measured spectra are shown in Figure 9. This corresponded well with the measurements with the impedance analyzer.

### 3.3. Transmit Experiments

Finally, transmission measurements were performed in immersion. For a DC voltage of 60 V and an AC voltage signal of 10 V (burst count 20), an uncoated CMUT chip (transmitter) and a coated CMUT chip (receiver) were mounted at a distance of 83 mm.

**Figure 10:**
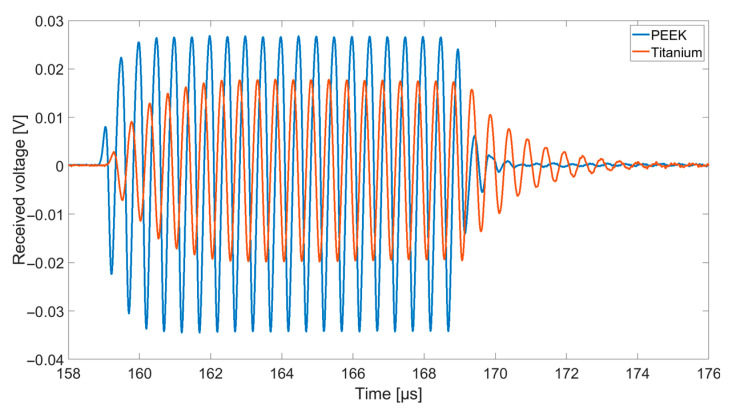
Received voltage signal at 2 MHz for coatings with 170 µm thick silicone and PEEK with a thickness of 25 µm as well as with 170 µm thick silicone with a titanium foil of 32 µm thickness.

Figure 10 shows an example of the received signals at an excitation frequency of 2 MHz for the receiving chip coated with a layer of silicone (thickness 170 µm) and a foil of PEEK or titanium.

**Figure 11:**
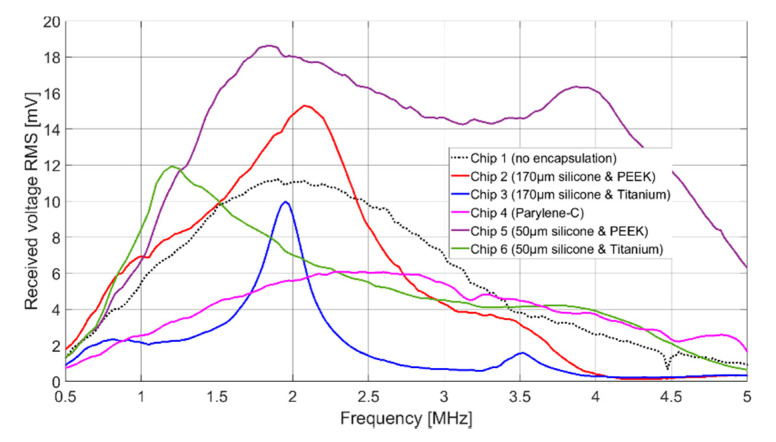
Receiving spectrum in Fluorinert with and without housing, 60 V_DC_, 10 V_AC_, sinusoidal burst count 20, distance 83 mm.

The resulting frequency spectrum of the receiving voltage is shown in Figure 11. As a reference, the black curve shows the spectrum for the uncoated CMUT Chip 1. Results can be found in Table 2.

### 3.4. Transmit Experiments Ex Vivo

**Figure 12:**
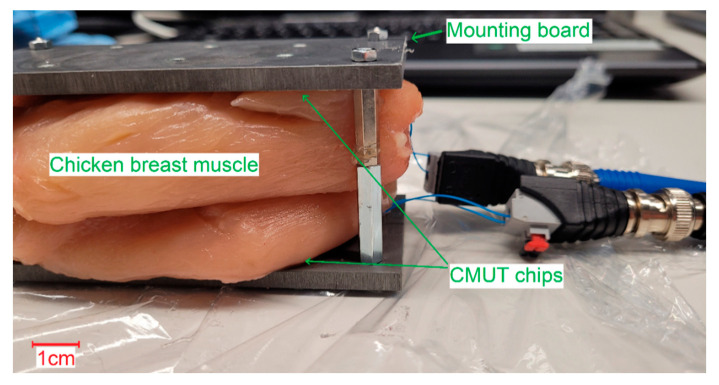
Transmission path for ex vivo experiments.

The experiment described below shows the functionality of the encapsulated ultrasound transducers in tissue. Using CMUT Chip 2 for transmitting and CMUT Chip 5 for receiving, first, ex vivo measurements were performed on chicken breast muscle (fillet) of 35 mm and 60 mm thickness. Multiple layers of chicken breast muscle were stacked on top of each other and bonded with ultrasonic gel to prevent transmission from being disrupted by trapped air. A photo of the experimental setup of the transmission path is shown in Figure 12. The CMUT chips were attached to the inner surface of the two boards. Compared to the previous transmit experiments (see Figure 5), the setup remained unchanged, only the Fluorinert was replaced by chicken breast muscle.

**Figure 13:**
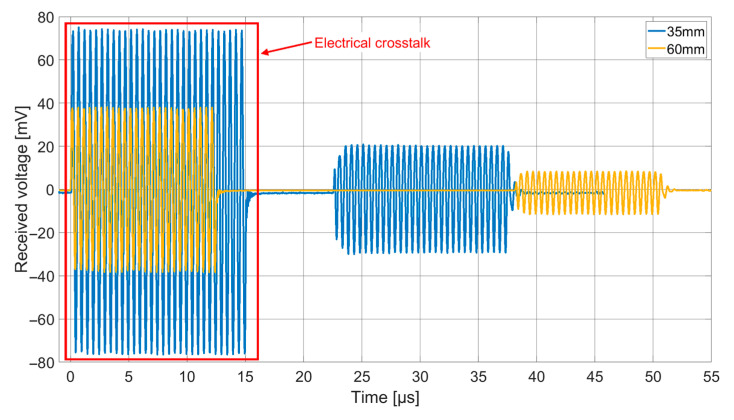
Received signal for the ex vivo transmit experiment at 2 MHz, Chip 2 (170 µm thick silicone and 25 µm thick PEEK) for transmitting, Chip 5 (50 µm thick silicone and 25 µm thick PEEK) for receiving different transmit depths, 60 V_DC_, 15 V_AC_.

The received voltage signal for an excitation frequency of 2 MHz and transmission paths of 35 mm, as well as 60 mm, can be found in Figure 13. The experimentally determined sound velocity of the meat was 1565 m/s, which was in good agreement with the value mentioned by Shishitani et al. for non-denatured chicken breast muscle (1569 m/s) [26]. For the transmission distance of 35 mm, the RMS of the receiving voltage was 17.8 mV, for a distance of 60 mm it decreased to 7.1 mV. This corresponded to an attenuation of 7.92 dB over a distance of 25 mm or an attenuation coefficient of 1.13 dB/(cm MHz). In the literature, the attenuation of ultrasonic waves in (beef) muscle tissue normal to the fibers was given as 1.1 dB/(cm MHz) [27], which matches the value found in the measurement presented here.

Thus, it could be shown experimentally that encapsulation with silicone and a PEEK foil is suitable for the use of CMUTs on muscle tissue and delivered clearly identifiable signals at least for distances of up to 60 mm.

**Figure 14:**
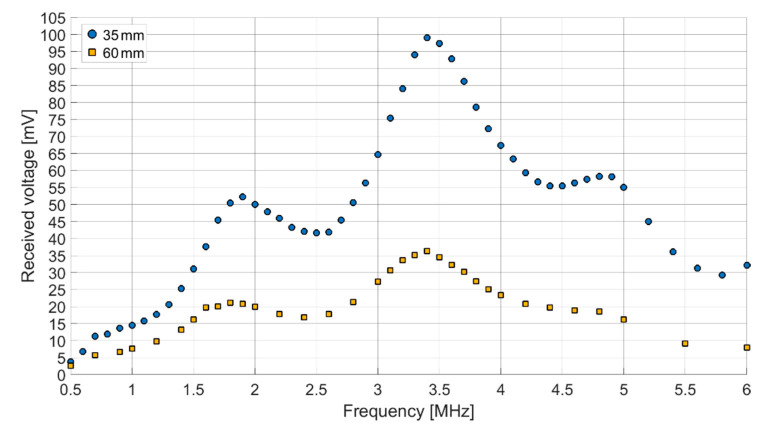
Receiving spectra of the ex vivo measurements, 60 V_DC_, 15 V_AC_, Burst count 20, Chip 2 (170 µm thick silicone and 25 µm thick PEEK) for transmitting, Chip 5 (50 µm thick silicone and 25 µm thick PEEK) for receiving distances of 35 mm and 60 mm.

The resulting receiving spectra of the ex vivo measurements for distances of 35 mm and 60 mm are shown in Figure 14. The main resonance frequency shifted to 3.4 MHz. Further maxima were found at 1.9 MHz and 4.9 MHz.

## 4. Discussion

The signal amplitudes for resonant excitation in Fluorinert FC-72, which can be found in Table 2, are for all cases of the same order of magnitude. Chip 2, Chip 5, and Chip 6 show even a higher signal amplitude than the uncoated CMUT chip (Chip 1). The higher signal amplitude of the two CMUT chips encapsulated with 50 µm silicone and a foil (Chip 5 and Chip 6) compared to the two CMUT chips encapsulated with 170 µm silicone and a foil (Chip 2 and Chip 3) can be explained as follows.

First, the ultrasonic signal is damped within the thicker silicone layer. A thinner silicone layer reduces this effect. Secondly, FEA has shown that the composite transducer consisting of CMUT membrane, electrode, silicone layer, and foil has maximum membrane deflections and thus maximum sound pressure at a silicone thickness of 60 µm (see Figure 6).

Comparing the results of Chip 2 and Chip 3 (and analogously for Chip 5 and Chip 6), the maximum signal amplitude is larger for a coating with PEEK than for a coating with titanium. One reason for this is the bigger difference in acoustic impedance between titanium (Z = 27.45 MRayl) and Fluorinert FC-72 (Z = 0.9 MRayl) compared to PEEK (Z = 3.04 MRayl) and Fluorinert FC-72. This discontinuity in the acoustic impedance causes a large part of the energy to be reflected at the titanium-Fluorinert interface. In addition, due to the higher Young’s modulus of titanium (E = 114 GPa) compared to PEEK (E = 3.76 GPa), a bigger force is required to achieve the same deflection.

In terms of bandwidth, Chip 5 gives higher values than all other CMUT chips. This is due to the fact that it was encapsulated with a silicone layer thickness of 50 µm, which is close to the thickness found to be optimal in FEA (t_ideal_ = 60 µm). A theoretical background of the results of the different encapsulations is currently being developed and will be part of a future publication.

A bandwidth of 122% (Chip 5) corresponds to a data rate of 1.85 Mbit with a coding of 1 bit per hertz. With a more conservative assumption of three pulses per bit, the achievable data rate is 0.617 Mbit. For applications that require a higher data rate, it can be significantly increased by using suitable control electronics and pulse coding. In addition to frequency coding of the signal, an 8-bit amplitude modulation is possible, which could increase the data rate by a factor of 8 to 4.93 Mbit. The significantly lower bandwidth for the CMUT encapsulated with 170 µm thick silicone and a titanium foil (Chip 3), can be explained by the higher acoustic impedance of the titanium foil, increasing the occurrence of interference between the foil and the CMUT membrane.

Both receiving spectra of the ex vivo measurements (Figure 14) show a frequency dependence of the signal amplitude, which differs from the results of the transmission measurements in Fluorinert FC-72. The objective resonance peak shifts from 1.85 MHz in Fluorinert FC-72 to 1.9 MHz ex vivo. However, the pressure being applied to the surface of the encapsulated CMUT chips when applying it to the muscle tissue emphasizes higher modes, so that the main resonance peak occurs at 3.4 MHz. While the original intention was to operate at a single frequency band at 2 MHz, this result, assuming appropriate electronics, gives the possibility to operate on two different frequency bands.

## 5. Conclusions

CMUT chips with a center frequency of 2 MHz in immersion were manufactured and characterized for encapsulation. Based on this, titanium and PEEK for a biocompatible housing, which was applied on the CMUT surface using a silicone coupling, were experimentally evaluated. In addition to titanium, the standard material for medical implants, PEEK was tested as a promising alternative. It was furthermore shown in FEA and experiments that the composite oscillator resulting from the encapsulation can be optimized in terms of bandwidth and signal amplitude by optimizing the thickness of the silicone layer. It could be shown that with the investigated encapsulation strategy, sufficient signal amplitudes and bandwidths can be achieved to transmit signals over 80 mm in Fluorinert FC-72 and over 60 mm ex vivo.

A necessary step towards actual application is the change from the laboratory electronics used in this work to integrated electronics powered by battery. Initial tests with prototypes have produced very promising results here.

In addition, in future work, the contact pressure to which the acoustic window of the implant is exposed inside the human body should be further investigated, as it alters the CMUT characteristics. Body temperature or movement through breathing could also have an influence here.

## Figures and Tables

**Table 1:** Material parameters used in the simulation.

Material	Density	V_long_	V_trans_
Silicone	7191 kg/m^3^	1000 m/s	167 m/s
PEEK	1285 kg/m^3^	2536 m/s	1086 m/s
Titanium	4480 kg/m^3^	6100 m/s	3100 m/s
Si	2350 kg/m^3^	8120 m/s	5200 m/s
Si_3_N_4_	3270 kg/m^3^	11,000 m/s	6250 m/s
SiO_2_	2650 kg/m^3^	5750 m/s	2200 m/s
Al	2690 kg/m^3^	6306 m/s	3114 m/s

**Table 2:** Results from the transmit experiments in Fluorinert for different encapsulations 60 V_DC_, 10 V_AC_, burst count 20, distance 83 mm.

	Maximum Voltage [mV]	Center Frequency [MHz]	−6 dB-Bandwidth
Uncoated	11.2	2.1	102%
170 µm silicone & 25 µm PEEK	15.3	1.8	78%
170 µm silicone & 32 µm titanium	10.0	1.9	21%
50 µm silicone & 25 µm PEEK	18.6	2.9	122%
50 µm silicone & 32 µm titanium	11.9	1.6	95%
5.5 µm Parylene-C	6.1	2.7	109%

## Data Availability

The data presented in this study are available on request from the corresponding author. The data are not publicly available because they are stored in Fraunhofer’s cloud. Access can be granted on request.
